# Prophylaxis and treatment of postoperative nausea and vomiting

**DOI:** 10.1016/j.bjae.2026.02.001

**Published:** 2026-03-11

**Authors:** A. Kovac, N. Choksi

**Affiliations:** University of Kansas Medical Centre, Kansas City, KS, USA

**Keywords:** antiemetics, postoperative nausea and vomiting, prevention and control, postoperative period

**Learning objectives**

By reading this article, you should be able to:

• State the incidence of postoperative nausea and vomiting (PONV), postdischarge nausea and vomiting (PDNV) in adults and postoperative vomiting (POV) in children.
• Describe management strategies to reduce the risk of PONV, PDNV and POV.
• List the different medications available for the prevention and treatment of PONV.
• Describe current management strategies using combination and multimodal antiemetic therapy for the prophylaxis and treatment of PONV.

**Key points**

• The goal for initial management of PONV is to identify and decrease the baseline risk of PONV.
• Adults and children should receive a prophylactic multimodal approach with combination of antiemetics from different drug classes, using the lowest effective dose.
• Important components of a multimodal approach used in enhanced recovery after surgery (ERAS) protocols include regional anaesthesia, opioid-sparing analgesics, adequate hydration and a combination of prophylactic antiemetics.
• Prophylaxis and treatment guidelines help to improve outcomes and decrease adverse events related to PONV.

Despite the implementation of multimodal pain management strategies and enhanced recovery after surgery (ERAS) protocols, postoperative nausea and vomiting (PONV) and postdischarge nausea and vomiting (PDNV) continue to be distressing problems and remain a challenge. After elective surgery, without prophylaxis, the overall incidence of PONV is 20–30%, and up to 70% in high-risk patients.^1^ A systematic review by Eberhart and colleagues reported a high rate of recurrence of emetic symptoms after a first episode in the recovery room after placebo or no treatment, suggesting the need for subsequent antiemetic treatment.^2^ For PONV (any symptoms of nausea or emesis), the recurrence rate was 65–84%, and for vomiting, 65–78%. Apfel and colleagues determined that PDNV may occur with a combined nausea and vomiting incidence of about 37% and persist up to 4–5 days after surgery.^3^ After the age of 3 yrs, PONV increases in children with a peak incidence of about 40% in the 11- to 14-yr-old age group. Before puberty, there were no apparent differences in PONV incidence between boys and girls.^4^

Without intervention, PONV and PDNV can lead to dissatisfaction, prolonged stay in the PACU and delayed discharge after ambulatory surgery. Adverse events associated with PONV can also cause unexpected hospital admissions, thereby increasing healthcare costs.^5^ The occurrence of PONV and PDNV can depend on the location of the health facility and whether interventions were used to reduce PONV or PDNV.^6^

PONV prevention can be achieved with risk assessment, baseline risk reduction and antiemetic prophylaxis. Patient, anaesthesia and surgery-related risk factors should be considered and have been covered in detail in another review.^7^ The goal of this review is to summarise current management for PONV prophylaxis and treatment in children and adults.

## Strategies for prophylaxis

During the preoperative workup, children and adult patients at high risk for PONV should be identified to establish a plan for prophylaxis against PONV and minimise adverse drug effects based on preexisting clinical problems such as prolonged QTc interval, Parkinson’s disease and closed angle glaucoma.^4^ Review of the patient’s electronic medical record (EMR), including reminders, can help identify patients at risk, and improve compliance with proper PONV management.^8^

### Reduction of baseline risk

**Fig 1:**
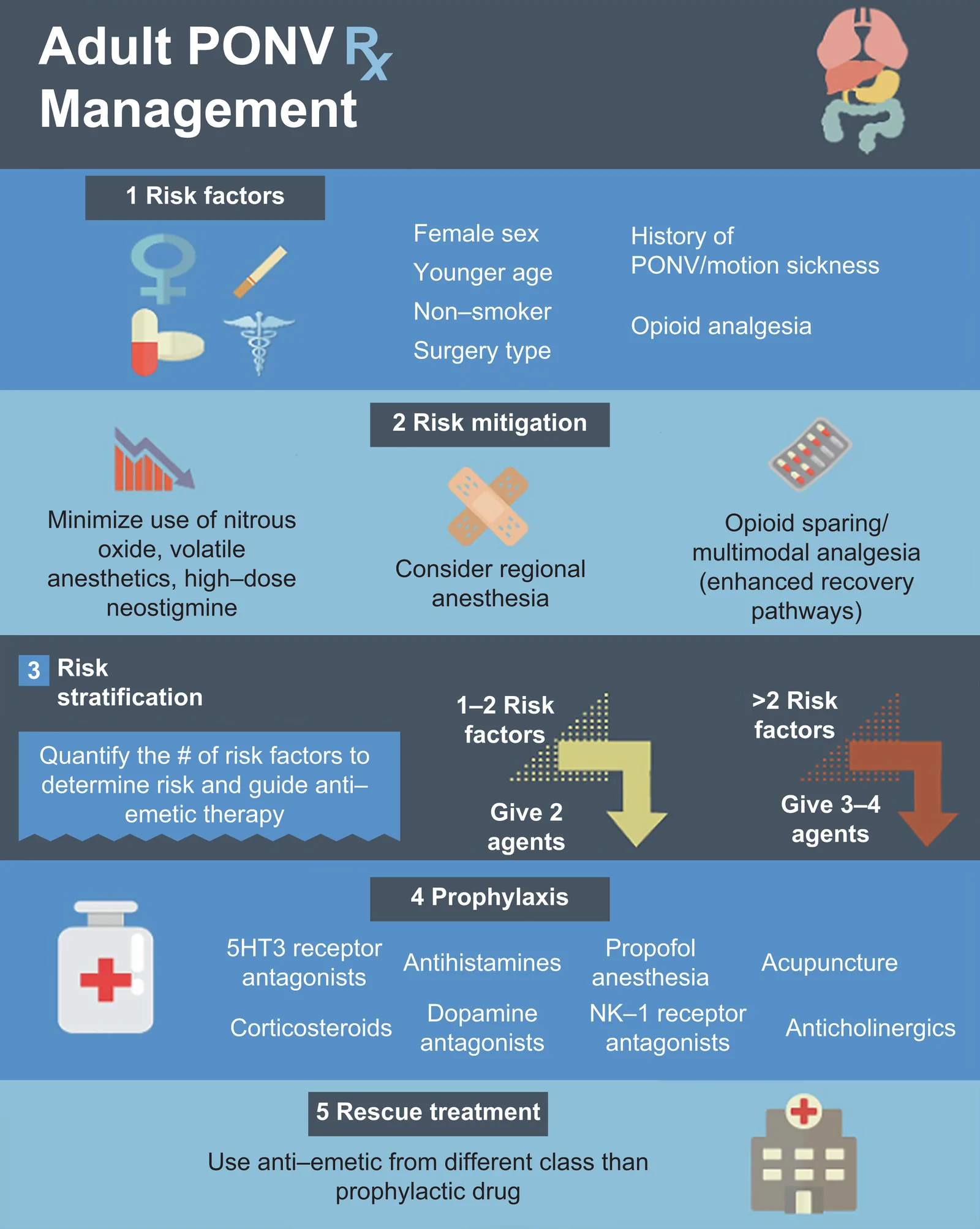
**Management of PONV in adults.** 5HT3, 5-hydroxytryptamine-3; NK1, neurokinin_1_; PONV, postoperative nausea and vomiting. Reproduced with permission from the American Society for Enhanced Recovery and Perioperative Medicine (www.aserhq.org).

**Fig 2:**
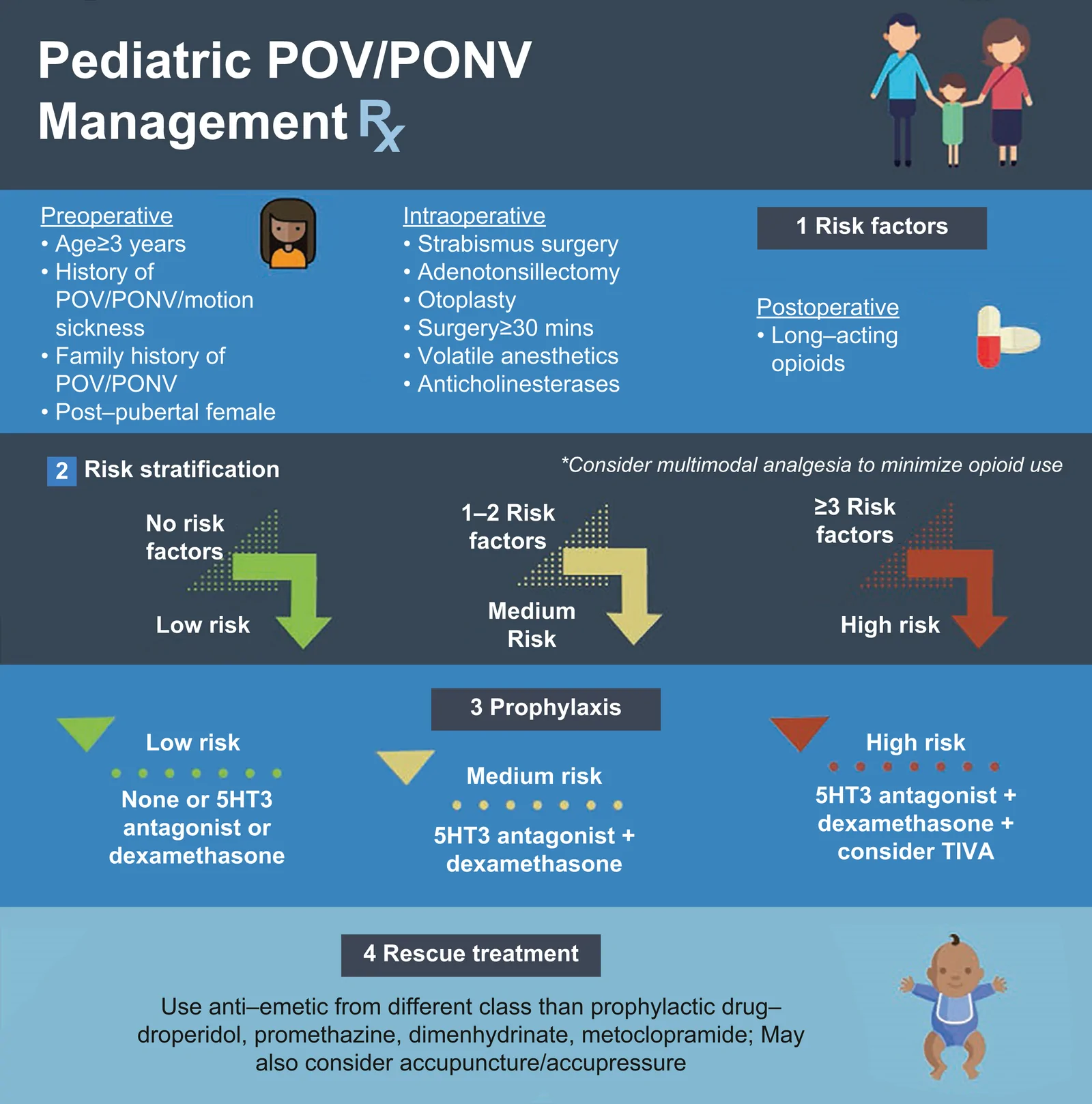
**Management of POV and PONV in children.** 5HT3, 5-hydroxytryptamine-3; NK1, neurokinin_1_; PONV, postoperative nausea and vomiting; POV, postoperative vomiting; TIVA, Total Intravenous Anesthesia. Reproduced with permission from the American Society for Enhanced Recovery and Perioperative Medicine (www.aserhq.org).

Strategies to help decrease the risk of PONV include using regional anaesthesia, and avoiding or minimising general anaesthesia (GA) and opioids by using multimodal opioid-sparing analgesic techniques (Table 1). If GA is necessary, a TIVA technique should be considered, using propofol for both induction and maintenance. When used in combination with other antiemetics, TIVA can help reduce PONV.^9^ Volatile agents, such as desflurane, sevoflurane and isoflurane, and gases such as nitrous oxide, should be avoided if possible as these agents stimulate receptors in the vomiting centre and chemoreceptor trigger zone.^10^ Opioids stimulate μ opioid receptors in the CNS, and peripherally in the gastrointestinal tract. A resulting delay in gastric emptying can cause nausea and vomiting. An approach that minimises opioids, using opioid-sparing analgesia with paracetamol and NSAIDS, should be used.^11^ Adequate i.v. hydration with colloids or crystalloids and vasopressor drugs such as ephedrine should be given to minimise hypotension.^12^ Using sugammadex, instead of neostigmine, for antagonisation of neuromuscular block should be considered as sugammadex has been associated with a lower incidence of PONV.^13^ Guidelines for the management of PONV prophylaxis and treatment are available for adults (Fig. 1) and children (Fig. 2).^4^

**Table 1:** **Strategies to reduce baseline risk of PONV.** GA, general anaesthesia. Reproduced with permission from Gan *et al.*^14^

Avoidance of GA by the use of regional anaesthesia
Use of propofol for induction and maintenance of anaesthesia
Avoidance of nitrous oxide in surgeries lasting >1 h
Avoidance of volatile anaesthetics
Minimisation of intraoperative and postoperative opioids
Adequate hydration
Using sugammadex instead of neostigmine for the antagonisation of neuromuscular block

## Antiemetic drugs

Prophylactic doses and timings for antiemetic drugs are listed in Table 2.

**Table 2:** **Antiemetic doses for prophylaxis and treatment of POV/PONV in adults and children**. PONV, postoperative nausea and vomiting; POV, postoperative vomiting. *Amisulpride: for treatment in adults, the dosage is 10 mg i.v.

Drugs	Adult dosage	Children dosage	Timing	Adverse effects
Ondansetron	4–8 mg i.v.	50–100 mcg kg^−1^ up to 4 mg i.v.	End of surgery	Headache, constipation, flushing, fatigue, malaise, raised liver enzymes
Granisetron	0.3 mg i.v.	40 mcg kg^−1^ up to 0.6 mg i.v.	End of surgery	
Ramosetron	0.3 mg i.v.	6 mcg kg^−1^ i.v.	End of surgery	
Palonosetron	0.075–0.25 mg i.v.	0.5–1.5 mcg kg^−1^ i.v.	After induction of anaesthesia	
Dexamethasone	4–10 mg i.v.	150 mcg kg^−1^ up to 5 mg i.v.	After induction	Transient increased blood glucose level
Droperidol	0.625–1.25 mg i.v.	10–15 mcg kg^−1^ up to 1.25 mg i.v.	After induction	Psychotomimetic effects, extrapyramidal adverse effects, sedation, lightheadedness, prolonged QT interval
Haloperidol	1 mg i.v.		After induction or treatment	
Aprepitant	40–80 mg p.o.	3 mg kg^−1^ up to 125 mg p.o.	1–2 h before induction	Headache, constipation, fatigue, decreased effectiveness of hormonal contraceptives
Fosaprepitant	32 mg i.v.		After induction	
	150 mg i.v.		After induction	
Scopolamine	Transdermal patch 1 mg every 3 days		Evening before surgery or in the preoperative period	Dizziness, dry mouth, visual disturbances, tachycardia, confusion, urinary retention
Metoclopramide	10–25 mg i.v.		15–30 min before the end of surgery	Sedation, hypotension (fast injection), headache, extrapyramidal symptoms
Amisulpride	5 mg i.v.*		After induction	Chills, hypokalaemia, hypotension, increased prolactin levels
Olanzapine	10 mg p.o.		Prior to induction	Sedation, transient visual changes
Dimenhydrinate	25 to 5 mg i.v.		Prophylaxis and treatment	Sedation, disorientation, dry mouth, drowsiness, cautious use in older
Promethazine	6.25 mg i.v.		Treatment	Caution of tissue injury if extravascular injection occurs
Prochlorperazine	5–10 mg p.o.		Treatment	Sedation, blurred vision, dizziness, difficulty urinating
Meclizine	25–50 mg p.o.		Prophylaxis and treatment of PONV, vertigo and motion sickness	Sedation, drowsiness, dry mouth, blurred vision

### 5-Hydroxytryptamine-3 receptor antagonists

First-generation 5-hydroxytryptamine-3 (5-HT_3_) receptor antagonists used for prophylaxis include ondansetron, granisetron and tropisetron.^15^ Ondansetron is commonly given for prophylaxis and treatment of PONV. Ondansetron 4 mg i.v. at the end of surgery has similar efficacy to dexamethasone 4–8 mg i.v. and haloperidol 1 mg i.v.^16^^,^^17^ Ondansetron is more effective than metoclopramide but less effective than palonosetron 0.075 mg i.v., aprepitant 80 mg p.o. or fosaprepitant 150 mg i.v. ^15^^,^^18^ Granisetron 0.3–1 mg is effective for PONV prophylaxis when given at the end of surgery.^15^ Tropisetron 2 mg i.v. is more effective than ondansetron 4 mg, but less effective than palonosetron 0.075 mg. Palonosetron, a second-generation 5-HT_3_ antagonist, has a half-life of 40 h and no effects on the QTc interval.^15^ With allosteric binding, positive cooperativity and receptor internalisation, palonosetron has 100 times higher affinity for the 5-HT_3_ receptor, and better efficacy than either ondansetron 4–8 mg 5-HT_3_ or dexamethasone 4–8 mg i.v.^19^ Ramosetron is a second-generation 5-HT_3_ antagonist with higher binding affinity, slower receptor dissociation and longer duration (6–9 h) than ondansetron (3–4 h). Ramosetron 0.3 mg i.v. has shown better efficacy than ondansetron.^20^

### Neurokinin_1_ receptor antagonists

Of the neurokinin_1_ receptor antagonists, aprepitant, available orally and i.v. as a prodrug (fosaprepitant), has a half-life of 40 h.^15^ A 40 mg oral dose has similar efficacy to palonosetron 0.075 mg i.v. and is more effective than ondansetron 4 mg.^21^^,^^22^ Aprepitant is useful in patients at high risk for PONV and PDNV, and cases where postoperative emesis is highly undesirable, such as neurosurgery and bariatric surgery. Recently, an i.v. formulation of aprepitant became available, given as a 32 mg dose for prophylaxis of PONV. Aprepitant and fosaprepitant increase metabolism of oestrogen and progesterone leading to lower levels of these hormones and decreased hormonal contraceptive efficacy.^4^

### Steroids

Dexamethasone 4–8 mg i.v. has anti-inflammatory and antiemetic effects and is given after induction of anaesthesia for prophylaxis.^23^ A review of the adverse effects of dexamethasone in surgical patients did not find an increased risk of postoperative infections. Caution is advised in diabetic patients who may be at increased risk for hyperglycaemia. However, a single dose has no effect on wound healing.^24^

### Antidopaminergic drugs

The antidopaminergic effect of droperidol has been shown to be effective for prophylaxis at doses of 0.625 mg (<1 mg) i.v. given at end of surgery.^4^^,^^15^ At this low dose, adverse effects such as prolongation of the QTc interval and akathisia were not a concern.^25^ However, a Food and Drug Administration (FDA) ‘black box’ warning exists for sudden cardiac death when doses higher than 25 mg are given.^25^ At the doses used for PONV, the potential for QTc prolongation was similar to ondansetron.^4^ Haloperidol 1 mg i.v. given at the start or end of surgery also has similar efficacy as the 5-HT_3_ antagonists with an adverse effect profile (QTc prolongation, sedation, akathisia) similar to droperidol. For treatment of PONV in the PACU, haloperidol had similar effectiveness, but more sedation compared with ondansetron 4 mg.^15^^,^^26^

Metoclopramide is a dopamine antagonist and gastric prokinetic medication occasionally used when other antidopaminergic medications such as haloperidol may not be available. For effective PONV prophylaxis, metoclopramide doses greater than 10 mg i.v. may be necessary. However, at high doses (25–50 mg), adverse extrapyramidal effects can occur.^15^^,^^27^

Amisulpride is an i.v. dopamine DA_2_/DA_3_ receptor antagonist approved for PONV prophylaxis (5 mg) and treatment (10 mg). Amisulpride is effective in treating patients in whom PONV prophylaxis has previously failed and is not associated with adverse effects such as sedation, QTc prolongation or extrapyramidal adverse effects.^15^^,^^28^

### Antihistaminergics

Histamine_1_ (H_1_) antagonists have been found to be useful for the treatment of nausea, vomiting and motion sickness. Diphenhydramine 25–50 mg i.v. is effective for PONV prophylaxis as a first-line medication.^15^ Phenothiazines are H_1_ antagonists with moderate muscarinic and dopamine (DA_2_) receptor blocking actions. Sedation is an adverse effect of this class of medications and should be used cautiously in older patients.^29^

### Anticholinergics

The anticholinergic scopolamine is effective for PONV prophylaxis when applied before surgery behind the ear as a transdermal (TD) patch the evening before or 2 h before surgery. It is also useful for high-risk patients with a history of motion sickness. Adverse effects because of its central anticholinergic action include dizziness and blurred vision, which are concerns, especially in older people.^30^

## Other interventions

Neuromodulator medications such as gabapentin and pregabalin have opioid-sparing effects that help to decrease PONV. They belong to a group of medications called gabapentinoids, which decrease the release of neurotransmitters that modulate pain, such as serotonin, glutamate and substance P. By decreasing the development of hyperalgesia, these medications help minimise opioid use. Preoperative gabapentin given at a dose of 600–800 mg p.o. and pregabalin 150 mg p.o. help reduce opioid consumption and PONV.^31^ However, adverse effects of sedation, visual disturbances and dizziness can occur. The FDA has issued a warning about the risk of respiratory depression, especially in older patients, when these medications are used in combination with other CNS depressants such as opioids.^32^

Olanzapine 10 mg is an antipsychotic medication used for chemotherapy-induced nausea and vomiting that has been found to be effective for PONV especially when used in a multimodal regimen with ondansetron and dexamethasone. Sedation and transient visual changes may occur.^33^

Midazolam is a short-acting benzodiazepine. Its anxiolytic effect has been shown to help reduce PONV when given as a 2 mg i.v. dose in combination with other antiemetics at induction of anaesthesia. Midazolam given at the end of surgery is not recommended because it can cause postoperative sedation.^34^

Propofol has antiemetic properties when used for maintenance of anaesthesia as part of a TIVA technique.^35^ Antiemetic properties (mean plasma concentration of 343 ng^−1^ ml^−1^) occur at a subhypnotic dose much lower than the concentration associated with GA (3–6 mcg ml^−1^) or sedation (1–3 mcg ml^−1^).^36^ A systematic review determined that a propofol bolus dose of 1 mg kg^−1^ followed by an infusion of 20 mcg kg^−1^ min^−1^ helped reduce PONV.^37^ When used as small (20 mg) i.v. doses in the PACU for rescue antiemetic therapy in patients in whom prophylaxis has failed, the effect of propofol has been shown to be brief, but as effective as ondansetron.^38^

Non-pharmacological options that have been reported with variable effectiveness include (i) acupoint pressure or stimulation at the 6th point of the pericardial meridian (PC6) for prophylaxis and (ii) chewing gum or aromatherapy (with peppermint, lemon or isopropyl alcohol) for the treatment of PONV.^4^

## Postdischarge nausea and vomiting

Opioids and volatile anaesthetic agents have comparable tendencies to cause PDNV. Several studies have shown that multimodal therapy is a key approach to prevent PDNV.^4^ Long-acting antiemetics such as promethazine, palonosetron, aprepitant, olanzapine, ondansetron oral disintegrating tablet (ODT), ramosetron ODT and TD hyoscine (scopolamine) patches have been effective in patients with a high risk of PDNV.^4^^,^^16^^,^^39^, ^40^, ^41^

## Combination therapy and multimodal approaches

As a prophylactic and multimodal approach to decrease the incidence of PONV and PDNV, combining antiemetics targeting different receptors has been found to be more effective than using single agents alone.^4^ This allows for improved PONV prophylaxis as a result of the synergistic effects, additive effects or both of each antiemetic working at different receptors. For PONV prophylaxis, the 5-HT_3_ receptor antagonists (i.e. ondansetron or palonosetron) are more effectively used in combination with dexamethasone.^42^^,^^43^ Oral aprepitant 40 mg in combination with ondansetron or dexamethasone is more effective than ondansetron or dexamethasone alone.^4^^,^^43^ Olanzapine 10 mg p.o. provides additional efficacy when used with ondansetron or dexamethasone.^33^ Apfel and colleagues^44^ determined that combining antiemetic therapy such as ondansetron plus aprepitant, or haloperidol plus dexamethasone, had a lower incidence of PDNV compared with single-drug prophylaxis. Adverse effects, including QTc prolongation and sedation, can be additive and are a concern when using multiple antiemetics in combination.^4^ More research is needed to evaluate the effectiveness of prophylactically combining three or more antiemetic medications for PONV or PDNV.

Opioids contribute to PONV. Opioid-sparing multimodal analgesia includes the use of NSAIDS, cyclooxygenase-2 (COX-2) inhibitors, acetaminophen (paracetamol), gabapentin and regional nerve blocks.^11^ A reduced need for opioid analgesics allows for a decrease in opioid-related adverse effects including PONV. Using ERAS protocols, multimodal management includes the use of anxiolytics (midazolam), limiting opioids (with the use of NSAIDS, COX-2s, gabapentin, paracetamol and nerve blocks), adequate i.v. hydration (at 20–25 ml kg^−1^) and use of double or triple antiemetic therapy (based on the number of Apfel PONV risk score factors). The PONV Consensus Guidelines recommends that patients with one or more PONV risk factors should receive prophylaxis using a multimodal approach with two or more antiemetics from different drug classes.^4^ Apfel and colleagues determined that when using a combination of two to three antiemetics, the incremental antiemetic benefit decreased with each added antiemetic as the baseline risk is further reduced.^44^ The choice of which combination antiemetic medication and dose to use for a particular patient group or surgery remains at the anaesthesia provider’s clinical judgement.

## Treatment of established PONV

If PONV occurs in the PACU, one should first rule out medication or mechanical (bowel obstruction) causes. Treatment should be based on the type of prophylactic therapy the patient has received. If PONV occurs within 6 h of receiving prophylaxis, one should not repeat the same prophylactic antiemetic but instead treat with an antiemetic from a different drug class. Studies have determined that repeating a 5-HT_3_ antagonist, such as ondansetron, in the PACU within 6 h of the first dose was no different in efficacy than giving a placebo. Combination therapy with medications of a different class is also more effective than treating with a single agent.^4^

If PONV occurs more than 6 h after the prophylactic antiemetic dose, one can treat with the same antiemetic used for prophylaxis, except for agents with a long duration of action such as dexamethasone, aprepitant, palonosetron or TD hyoscine. Haloperidol 1 mg i.v. or amisulpride 10 mg i.v. can also be used for rescue treatment. If no prophylaxis antiemetic was given, it is recommended to begin treatment with a 5-HT_3_ antagonist such as ondansetron 4 mg i.v. A bolus dose of propofol 20 mg i.v. also has been shown to be a short-duration rescue treatment for PONV in the PACU.^34^ A bolus dose of propofol 10 mg i.v. was less effective than droperidol 1.25 mg.^4^

For use in treatment of PONV or PDNV, promethazine is available in i.v., oral or i.m. formulations. Slow i.v. (6.25 mg) or deep i.m. (25 mg) injection can be used for treatment of motion sickness or PONV. However, caution should be exercised in patients with seizure disorders. If inadvertent s.c. or intra-arterial injection occurs, severe tissue necrosis can result. Although promethazine is not recommended as a first-line medication for PONV prophylaxis, it can be considered as a rescue antiemetic, especially in patients in whom prophylaxis has failed. Although histamine (H_1_) antagonists may be useful for treatment, they can cause sedation.^15^

## PONV in children

The approach to managing PONV in children is similar to that used in adult patients. Risk factors for PONV in children have been previously discussed previously in this journal.^7^ Common emetogenic surgeries in children are adenotonsillectomy and strabismus surgery. Baseline risk should be decreased, and prophylactic antiemetics given. Dexamethasone 0.15–1.0 mg kg^−1^ given at induction of anaesthesia has been shown to be effective in decreasing early and late PONV on the first postoperative day, and pain on the second day. Children with three or more risk factors should receive at least two antiemetics.^4^

Combination antiemetic therapy using ondansetron 150 mcg kg^−1^ plus dexamethasone 250 mcg kg^−1^ has been an effective method for preventing PONV.^40^ Granisetron 40 mcg kg^−1^ i.v., palonosetron 0.5–1.5 mcg i.v. or ondansetron 150 mcg i.v. is equally effective for PONV prevention in strabismus surgery. For children at low risk, no prophylaxis or single therapy with a 5-HT_3_ antagonist is recommended. For medium-risk children, double therapy with a 5-HT_3_ antagonist plus dexamethasone is the method of choice.^45^ Combination antiemetic prophylaxis with a subhypnotic propofol infusion plus dexamethasone has been shown to be more effective than dexamethasone alone in children after tonsillectomy.^46^ For high-risk children, triple antiemetic therapy with a 5-HT_3_ antagonist plus dexamethasone plus a TIVA technique is recommended^4^ (Fig. 2).

## Conclusions

Despite the introduction of new antiemetics and anaesthesia techniques, PONV continues to be a complex problem in adults and children, with many different causes and risk factors. The goal for initial management is to identify and decrease the baseline risk of PONV. To reduce the risk, all adults and children at risk should receive a prophylactic multimodal approach. To minimise the possibility of adverse effects, combination prophylactic antiemetics should be chosen using the evidence-based lowest effective dose from different drug classes. Opioid-sparing analgesic regimens, use of regional anaesthesia, adequate hydration and combination of prophylactic antiemetics are important components of a multimodal approach and frequently used in ERAS protocols.

## MCQs statement

The associated MCQs (to support CME/CPD activity) are accessible at www.bjaed.org/cme/home for subscribers to *BJA Education*.

## Declaration of interests

The authors declare that they have no conflicts of interest.
